# Citizenship, Vulnerability and Mental Incapacity in England, 1900–1960s

**DOI:** 10.1017/mdh.2019.27

**Published:** 2019-06-18

**Authors:** Janet Weston

**Affiliations:** Centre for History in Public Health, London School of Hygiene & Tropical Medicine, 15-17 Tavistock Place, London WC1H 9SH, UK

**Keywords:** Law, Psychiatry, Court of Protection, Lunacy Office

## Abstract

Over the twentieth century, the Lunacy Office (renamed the Court of Protection in 1947) was responsible for appointing ‘receivers’ to manage the property of adults in England who were found incapable of managing their own affairs. Tens of thousands of people were in this position by the 1920s, and numbers continued to grow until after Second World War. This article uses the archives of the Office to examine the evolution of the concept of mental incapacity over the first half of the twentieth century, offering a corrective to the popular impression that the time before the Mental Capacity Act of 2005 was an era of ignorance and bad practice. It examines the changing ways in which being ‘incapable’ was understood and described, with particular reference to shifting ideas of citizenship. I argue that incapacity was not always seen as absolute or permanent in the first half of the century, that models of incapacity began to include perceived vulnerability in the interwar period and that women in particular were seen in this way. From the 1940s, though, the profile of those found incapable was changing, and the growing welfare state and its principles of employment and universality saw the idea of incapacity narrowing and solidifying around knowledge deficits, especially among the elderly. This brings the history of the Lunacy Office into the twentieth century and connects it to current concerns around assessments of mental capacity today.

In the summer of 1939, as Europe hovered on the brink of war, a smaller drama unfolded in the Dorset village of Chilfrome. Since the death four years earlier of the local general practitioner, Dr Norton, neighbours had viewed the goings-on at his house The Old Rectory with misgivings. Dr Norton had left his substantial estate – including The Old Rectory – in trust for his housekeeper, Miss Beatrice Alexander, but she was soon joined in residence there by the ill-reputed Humphries family. None of the Humphries adults appeared to be employed, and excessive drinking was rumoured to be their preferred pastime, but who was paying for this lifestyle? What was becoming of Miss Alexander, a timid and nervous lady approaching sixty years of age, and the comfortable retirement provided by her late employer? Following a particularly unpleasant argument at The Old Rectory, after which he was told never to return, a close friend of Dr Norton finally wrote to a lawyer of his acquaintance in despair. ‘Miss Alexander’s trouble is weakness of character chiefly,’ he explained. ‘Still she is of low mentality and I do not think your mental expert would have any doubt after interviewing her that she was quite unable to manage her own affairs. Do something if you possibly can. The position of this poor weak lady is really deplorable.’^1^

His correspondent was uniquely well positioned to act. Assistant Master Ronald Poyser was one of those responsible for the appointment of ‘receivers’ for individuals in England and Wales who were found to be ‘of unsound mind and incapable of managing himself and his affairs’.^2^ Receivers assumed full responsibility for the individual’s property and finances for the duration of their mental infirmity and were overseen by the Office in Lunacy at the Royal Courts of Justice. Once alerted to Miss Alexander’s situation, the Office quickly gathered information about her property, her family and her living situation, and received a number of statements from those who had met her. Miss Alexander was eventually deemed incapable of managing her affairs, and the Official Solicitor was appointed as her receiver in July 1939.

Miss Alexander’s ability to manage her own affairs – her mental capacity – was assessed, formally and informally, by doctors and lawyers, friends and acquaintances. She was one of over 20 000 individuals whose estates were in the hands of receivers in the late 1930s, a figure which had more than doubled since the early 1920s and would continue to rise in the decades to follow.^3^ The archives of the Official Solicitor and the Office of the Master of Lunacy, renamed the Court of Protection in 1947,^4^ contain a 2% sample of case files from the early 1900s to the 1970s. These files include medical statements, official reports and extensive correspondence between the Offices, their experts, receivers and those like Miss Alexander who were found incapable. The 244 cases currently open for inspection form the basis of this article, offering a glimpse into the evolution of the medico-legal concept of mental incapacity in England over the first half of the twentieth century.

I pursue three interlinked strands of argument. The first offers a corrective to an impression that the time before the Mental Capacity Act of 2005 – the first piece of legislation for England and Wales to refer to ‘mental capacity’ specifically – was an era of ignorance and insensitivity. In the words of one consultant, for example, prior to this Act

many people were written off, whether or not they had a learning disability or dementia or a diagnosis such as schizophrenia, it was just assumed they can’t make decisions themselves, they’re provided with institutional care and have all their independence and autonomy removed.^5^

Many of the judges nominated to sit in the Court of Protection following the Mental Capacity Act reportedly shared this view of the Court’s history and assumed that all pre-2005 case law and practice should be wholeheartedly cast aside.^6^ This impression has been encouraged by a silence within historical scholarship regarding mental capacity decisions over the twentieth century, although research into earlier periods has signalled their potential.^7^ By looking more closely at decisions from the early twentieth century through to the 1960s about who was incapable of managing their affairs, I show that there was a much more nuanced approach than is generally understood. Some individuals, certainly, were ‘written off’, but until the post-Second World War period, generalisations were often resisted and bespoke solutions crafted. Ideas of mental incapacity were also constantly changing, which connects to my second strand of argument.

This second strand builds upon Peter Bartlett’s work on lunacy inquisitions in the nineteenth century, which has suggested some key shifts in how the concept was understood. Bartlett has argued that nineteenth-century assessments of an individual’s capability to manage their own affairs focused on the presence or absence of delusions, but by the late 1900s, this had been replaced by attention to the individual’s reasoning ability and intellect.^8^ I consider this shift in more detail, suggesting how it came about. I go on to show that it is not the whole story. From the 1920s to the 1940s, in deciding whether someone like Miss Alexander was capable of managing her affairs, those around her at the Lunacy Office were not only considering delusions or reasoning ability. They were also willing to think about her vulnerability. This took into account individual social circumstances and personality: in the name of preventing individuals who were perceived as vulnerable from making decisions that were not authentically theirs, the boundary between being fully capable and fully incapable was blurred. I examine three contrasting cases in some detail to illustrate these points, and I present some ideas as to why this attention to vulnerability emerged and then disappeared.

These explanations form the third strand of argument. I follow historians and other scholars of disability and gender by thinking about how shifting models of citizenship are reflected in social policy or, in this case, medico-legal practice.^9^ This moves away from the preoccupation of many older histories of mental health law, and medical law in general, with the balance of power between law and medicine.^10^ The swing of the pendulum between ‘legalism’ and ‘medicalism’^11^ is, I suggest, less useful here than the idea of citizenship. I argue that considerations of an individual’s capability to manage their finances were informed by the extent to which different types of person were viewed as full citizens, particularly in relation to age, gender and, to a lesser extent, class. I also argue that the expansion of the welfare state in the 1940s and its principle of universality had a significant impact on this, creating an era of change in the late 1940s and 1950s. Specifically, and perhaps counter-intuitively in light of scholarship on the ‘psy’ disciplines that portrays expansion and increasing surveillance throughout the twentieth century,^12^ I identify a retraction or shrinkage in Court of Protection interventions in the decades following the Second World War: factors for assessing incapacity narrowed, those deemed incapable became more homogenous and the boundary between capacity and incapacity hardened.

This article touches throughout on ideas currently prevalent in critical legal scholarship on mental capacity law. A binary approach to in/capacity is one such concern, as is the position and perception of vulnerability.^13^ This scholarship often acknowledges the value of historical perspectives but to date has not looked beyond the origins in the 1980s of current mental capacity legislation. The longer history presented here demonstrates the unspoken and unacknowledged role played by vulnerability and the importance of ideas of citizenship to mental incapacity in the past. It also shows that the ideas underpinning mental incapacity can, and do, change.

## 1 Certification, Incapacity and Being ‘Written Off’

Let us begin with the impression that individuals were often ‘written off’ prior to the Mental Capacity Act of 2005. Were those with certain diagnoses always seen as incapable of managing their affairs, and did they have their autonomy removed as a result? The answer to this is equivocal. In the early twentieth century, those detained in hospitals as persons of unsound mind were often also assumed to be incapable of managing their affairs, irrespective of their illness or state of mind. Yet this was neither constant nor absolute. The correlation between hospitalisation and a lack of capacity was at its strongest in the 1910s and 1920s, but even then the Lunacy Office and others were sometimes reluctant to define such patients as incapable. By the 1950s, the assumption that patients in mental hospitals were incapable of managing their property was under attack. Furthermore, the Office readily considered recovery, and some patients had full control over their property returned to them; still more remained the subject of a formal receivership but made some decisions about their own affairs. All this indicates that ideas of incapacity were changing, that it was acknowledged as potentially transient and that there was not always a hard boundary between being capable and incapable.

At the beginning of the twentieth century, most of those found incapable of managing their affairs were also certified and detained as persons of unsound mind. It is worth clarifying the two strands of mental health law that generated these distinct legal states. Admittance to asylums was governed by Part 1 of the 1890 Lunacy Act, which required two medical certificates and the signature of a ‘judicial authority’ for reception and detention in an institution. This came to be known informally as being ‘certified’ and was overseen by Commissioners in Lunacy and the Board of Control. Subject to regular revision, it was adjusted by new mental health legislation in 1930 and 1959.

Applications for a person to be found incapable of managing their property and affairs, on the other hand, were the responsibility of the Lord Chancellor. In accordance with Parts 3 and 4 of the 1890 Lunacy Act, the Judge and Masters in Lunacy had jurisdiction over the property of any person, as long as it was proved to their satisfaction that ‘such person is through mental infirmity arising from disease or age incapable of managing his affairs’.^14^ It was by fulfilling this criterion, which remained almost unchanged throughout the twentieth century, that individuals were found to lack capacity and receivers were appointed to manage their money.

Receivership applications usually came from a relative and were prompted by the need to pay hospital bills or deal with an inheritance, tenancy or other financial matter. Applicants had to supply full details of the person’s family and fortune, along with a recent medical affidavit confirming that the individual was indeed incapable. These affidavits typically contained some diagnostic and descriptive information about the nature of the person’s illness, to support the doctor’s conclusions: representative examples from the early twentieth century include a diagnosis of epilepsy with mania leading to ‘attacks of acute excitement during which he is incoherent in speech, violent, and destructive and uncontrollable’^15^ and ‘mania’ manifested in quarrelsome, troublesome conduct, irritating other patients, telling lies and producing ‘imaginary grievances’ and exaggerations.^16^

These statements were similar to those accompanying petitions for certification and were readily accepted as sufficient evidence of incapacity to manage one’s affairs. Although the archives do not contain any files relating to unsuccessful applications for receivers, it was relatively rare for there to be any hesitation over the incapacity of someone already certified and detained as a person of unsound mind. This apparent correlation between certification and incapacity was supported by case law from the early 1900s, which began to suggest that a certified person could never validly execute a deed or enter into a contract.^17^ Hospital policies adopted the same line, prohibiting certified patients from signing any document. Family members were often told in no uncertain terms that their relative’s signature was ‘not valid on any legal document’.^18^ Although some were taken aback by this, others seemed to share the impression that even those who seemed ‘quite sane’ in many respects would not be able to make valid decisions about their affairs if they were certified.^19^ The Lunacy Office itself encouraged this, not only by agreeing with hospital policies when approached for a second opinion but by changing its guidelines for applicants. As its workload increased in the 1930s, it ceased to require medical evidence of incapacity when the individual in question had already been certified.^20^ The unstated explanation for this was, presumably, that the fact of being certified was evidence enough.

Yet, as this change implies, being certified had not always meant that an individual was viewed as incapable of managing their affairs. Separate medical evidence addressing incapacity *had* previously been required. According to the authoritative textbook on the subject from the 1870s, it was ‘presumed that the Lord Chancellor would require some evidence of the character of the insanity’ in applications relating to those detained in asylums, and an inquisition would then be held to consider the question of incapacity in full.^21^ Where smaller amounts of property were involved, the quicker and cheaper process without inquisition required a report to persuade the Lord Chancellor that the individual was incapable, irrespective of whether the individual was in an asylum. ‘The affidavit of one medical man (physician, or surgeon) may generally be sufficient,’ this guidance noted, but more could be sought if necessary.^22^ In the nineteenth century, case law was also clear, for example, that a ‘confirmed lunatic’ could execute a deed as long as they were lucid at the time.^23^ A Royal Commission summoned in 1906 to investigate ‘feeble-mindedness’ was equally clear that ‘the certified lunatic might be capable of managing his property’.^24^

This was sometimes forgotten over the following decades, as another Royal Commission discovered in the 1950s. This later Commission protested in its 1957 Report (known as the Percy Report, after the Commission’s chair) that current thinking was muddled, and placed the blame upon the 1890 Lunacy Act, which ‘seem[ed] to assume incapacity’ among those already certified.^25^ The 1890 Act simply affirmed that the Lunacy Office had jurisdiction over certified persons without any further comment, whereas it expressly stated that the Office would have to be convinced that anyone else was incapable before taking action. This implied that it was not necessary to convince the Office that a certified person was incapable. This did not reflect administrative policy immediately before or after 1890 and its implications may have been accidental, but it was nonetheless influential. Also discouraging closer enquiry into the capabilities of certified individuals was the therapeutic pessimism of the early 1900s and a notion that the process of asylum admissions already set a high bar and was carefully monitored and reviewed. Paradoxically, the processes introduced in 1890 to assuage Victorian anxieties over sane persons institutionalised by cruel relatives and amoral doctors may have introduced a strong impression that those actually certified after this time were extremely and undoubtedly mad.

Challenges to the assumption that certified patients were invariably incapable of managing their affairs came from the growth of voluntary treatment for mental illness in the interwar period. Increasing numbers of voluntary patients, whose legal status was unaffected by their diagnosis or hospitalisation, raised questions and practical difficulties about the position of certified patients. Early and voluntary treatment for a wider variety of mental ailment was itself part of a shift towards seeing mental health as a spectrum, without firm boundaries. Such a shift was embodied in the 1930 Mental Treatment Act and anticipated by the Mental Deficiency Act of 1913. The attempt of the latter to define borderline states of ‘feeble-mindedness’ was indicative of interest in grey areas and abnormal states of mind that did not prevent everyday functionality.^26^ Such abnormal states would not necessarily warrant detention in hospital, but they did present challenges to ideas of criminal responsibility and self-governance.^27^ Evidence feeding in to the Mental Health Act of 1959 sought to eliminate assumptions about who lacked capacity to manage their own affairs, as did the Act itself.^28^ By the 1960s, thanks to this and the beginnings of deinstitutionalisation, less than half of those on the books of the Court of Protection were also certified and detained in hospital.^29^

Even before the 1960s, though, not all certified individuals, nor all those on whose behalf the Lunacy Office acted, were ‘written off’. The Percy Report cited evidence that, irrespective of formal guidelines, applications for the appointment of a receiver *always* included medical evidence,^30^ and the archives bear this out. Indeed, cases relating to individuals detained in hospital were sometimes delayed while the Office waited for such evidence.^31^ Although a template form for medical evidence was introduced in the 1930s and the information entered onto it was occasionally very brief, it was not as a rule markedly different from earlier medical evidence in length or quality.^32^ In practice, then, the Lunacy Office would not appoint a receiver on the basis of certification alone but required some additional confirmation of incapacity from a doctor.

Hospitals and the Lunacy Office would also sometimes use their discretion, or find ways of working around the system, if they were doubtful that an individual was truly incapable. The Percy Report gave an example of ‘a case in which a certified patient was discharged a fortnight after admission in order to deal with the essential business of his small-holding, in spite of the fact that his hospital treatment was not finished’. He was then re-certified once the business was complete.^33^ This suggests that those involved felt that the patient was mentally quite able to deal with the small-holding, despite his illness, and the only hurdle was his perceived legal status as a certified patient. Similarly, when a long-term patient at the County Mental Hospital in Chester, Ada Grundy, inherited £100 from a relative, the Medical Superintendent was clear that Miss Grundy *could* sign a receipt for the funds and he would be prepared to act as a witness to her signature.^34^

For its part, the Lunacy Office was inclined to hesitate to intervene if they received some indication that a certified patient was actually or nearly capable. Mrs Ada Gidney, for example, was certified in 1917 following the deaths of three of her four children but was said to be ‘only suffering from depression’. The Lunacy Office requested a report from one of its specialists, a Lord Chancellor’s visitor,^35^ who gave an equivocal assessment: Mrs Gidney was ‘not at present able to resume the management of her affairs and I am somewhat doubtful as to the likelihood of trustworthy[ness] in the near future’, but on balance, ‘it would be well to give her a chance’. The Office postponed its decision, acknowledging that Mrs Gidney’s capacity was unclear and likely to change over time.^36^ Anna Lockett’s case was handled similarly.^37^ In the case of Arthur Middleton, in the early 1950s, the Court declined to act for over two years. Mr Middleton’s doctor initially confirmed that ‘although Certified [he] is capable of managing his own affairs’, and Mr Middleton himself wrote to protest the proposed receivership, asking ‘to be allowed to return home to attend to my own affairs and i [sic] am big and strong enough to do so’. The Court took no action until it had received several medical statements to the effect that he was incapable, and even then it appeared reluctant.^38^ In practice, the Office was loath to declare someone incapable if their infirmities seemed transient, the patient was unwilling and their affairs were not urgent.

Office policy and practice also acknowledged that patients might recover and resume management of their affairs. This echoed a centuries-old recognition that ‘lunacy’ was not necessarily permanent. Victoria Longhurst and Obadiah Hopkins were both restored to their property shortly after release from hospital.^39^ Robert Gladstone’s receiver was in place for only a matter of three months, in light of his speedy recovery.^40^ Arthur Short and Jean Carr, whose cases will be considered in more detail below, both grew increasingly dissatisfied with their receivers and eventually applied successfully for their discharge.^41^ In the cases of individuals such as these two, who had receivers but were not certified, the Lunacy Office asked the Lord Chancellor’s visitors to consider whether the receivership was still necessary, and it would sometimes ask receivers as well.^42^ Undoubtedly, for those who remained hospitalised, who could not find medical support for their desire to resume control over their affairs or who could not navigate the necessary bureaucracy, challenging the status quo was all but impossible. Some were hugely frustrated by these barriers.^43^ Nevertheless, throughout the first half of the twentieth century, mental incapacity was *not* understood to be a permanent and hopeless state.

Even where receivers remained in place, patients were sometimes granted some control over their affairs. This was less likely for those who were hospitalised, where institutional policies and regulations presented practical difficulties, but even then some patients were given opportunities to go into town, for example, or to stay with friends for a few weeks, and many were free to spend a weekly allowance as they chose. Frederick Fitch spent his money in the local pub.^44^ Patients living outside of hospitals often had considerable independence, taking employment, entering into tenancies and managing their own income and expenses. Francis Raggett supplemented the money paid to her by her receiver by making and selling knitted goods, while Arthur Sims and Arthur Short held jobs and did not have to account to anyone for their earnings.^45^ Others were consulted about financial decisions by receivers and by the Lunacy Office itself.^46^ The rationale for these arrangements was very rarely specified but seems to have been based on a combination of factors, including a wish to avoid disputes, a sense that having some control could improve mental well-being and mental capacity itself, a fairly deeply embedded desire to respect the wishes of those with receivers where possible^47^ and a nuanced view of incapacity as being more complex than the blunt instrument of the receivership and the letter of the law allowed.

## 2 Changing Patients, Changing Views

Over the twentieth century, the ‘certified’ ceased to dominate the work of the Court of Protection. The roots of this shift were in section 116 (1) of the 1890 Lunacy Act, which had given the Lunacy Office jurisdiction over the affairs of anyone at all as long as they were deemed incapable through ‘mental infirmity arising from disease or age’. Disease and age were undefined in the Act and interpreted loosely.^48^ This provision was intended to cover those who were not ‘lunatics’ but were enfeebled in some other way and in need of protection from abuse – those who were ‘liable to be robbed by anyone’, as the author of the 1890 Act and later Master in Lunacy put it.^49^ Foreshadowing more recent ‘nervousness about perceived paternalism and interference in the lives of individuals’,^50^ he argued that it was preferable, on balance, to be able to protect the weak from hardships and exploitation, even if it raised questions about individual liberty in difficult cases. This provision was reportedly popular from the outset.^51^ Alongside reductions in the cost of making receivership applications, it gradually changed the types of person with whom the Lunacy Office was involved.

This changing patient profile affected and was affected by the ways in which being incapable of managing one’s affairs was perceived. ‘Mental capacity’ had no technical meaning or legal test until the 1990s, and being ‘incapable to manage one’s affairs’ remained undefined until 2002.^52^ Prior to this, the general principle followed by the courts whenever capacity was contested was that it ‘is the ability (whether or not one chooses to exercise it) to understand the nature and quality of the relevant transaction’ or act,^53^ but the Lunacy Office did not explicitly refer to this in any of its files or handbooks. Its determinations of incapacity were not formally appealed over this period, meaning there are no judicial statements as to how its decisions should be reached. Those involved in interventions were therefore left to draw upon personal and shared views of what exactly this meant, and these views suggest trends in the way that incapacity was assessed and understood.

In the early twentieth century, the Office’s patients were typically described as suffering from mania, dementia or melancholia, usually with delusions or hallucinations. This chimes with Peter Bartlett’s view that assessments of capacity in the nineteenth century were based on delusion or ‘the correct perception of facts at the basis of a decision’.^54^ In their affidavits, medical superintendents described certified patients as confused and forgetful, incoherent or rambling in their speech, with episodes of unresponsiveness, noisy excitement or destructive violence. Specific delusions were often described. Frank Fallows, for example, ‘expressed himself as under the guidance of a certain “underground system of lines” which is in the hands of some people called “The Talkers”’. Clara Picton heard disturbing instructions from God’s voice in her stomach. Emily Laird and Emma Tatlock each gave a common complaint, Miss Laird reporting that she was tormented by electricity and Miss Tatlock that she was persecuted by people around her, both visible and invisible. Delusions of fatal illness, destitution or serious wrongdoing were also regularly reported, particularly among those who were described as ‘dull’ or ‘depressed’.^55^ A general loss of self-management and self-control was also often described, with notes that patients were unable or unwilling to dress or keep themselves clean and well nourished, were ‘wet in habits’ or were prone to extremes of emotion, from wild laughing or weeping to bursts of foul language and anger. These false beliefs and confusing behaviours were evidence in and of themselves of an incapability to manage one’s affairs.

Alongside these, the absence of particular knowledge or skills began to emerge as signs of incapacity. This marks the beginning of Bartlett’s shift to a focus on intellectual ability, or ‘a standard of reasoning capability requisite to reach judgements’.^56^ This is first apparent in assessments of those deemed mentally defective or imbecile. A number of historians have explored the interest in mental defect in the late nineteenth and early twentieth centuries, and the introduction of four classes of mental defect in the Mental Deficiency Act of 1913.^57^ The categories of ‘feeble-mindedness’ and ‘moral imbecility’ tested the boundaries of the concept, arguably encompassing many more people than would previously have been considered abnormal. The 1913 Act allowed all those so found to be placed in institutions or to have guardians appointed. As Mathew Thomson has argued, this was an exclusion from the rights and responsibilities of citizenship, made conceivable because mental defect was seen as a fundamental biological difference.^58^ Almost immediately, the Lunacy Office began to receive applications for the appointment of receivers for those who were ‘defective within the meaning of the Mental Deficiency Act 1913’.^59^

These applications, where mental defect as opposed to other forms of illness or disability is described, give a picture of variable capabilities and difficulties among individuals, although they change little over time. William Sims was unable to read, write, answer any questions, keep himself clean, tell his age or name or ‘appreciate any remarks that are made to him’, and neither Blanche Parrish nor Leslie Trouson could wash or dress themselves or communicate at all in writing or verbally.^60^ Communication skills and the ability to take care of oneself were key. Where the ‘mentally deficient’ demonstrated adequate abilities in these fields, doctors would comment upon their knowledge of money matters. William’s brother Arthur Sims could read and write a little and knew that ‘twelve pence make a shilling and that there are twenty shillings in a pound’, but his arithmetic was extremely poor and he had ‘never heard of investment, per cent, interest’ or ‘having a stamp or receipt’ after paying a bill.^61^ Additional warning signs for incapacity were a lack of independent thought and self-control. It was here that Annie Alpass and Thomas Scotney were found wanting, Miss Alpass because she appeared ‘not capable of forming her own opinions, but will do what she is told by anyone relation or not’ and Mr Scotney for being ‘devoid of initiative’ and requiring instruction in all things.^62^ This attention to autonomy also provided an entry point for considerations of vulnerability, as the next section will discuss.

There are, though, only fifteen cases of patients diagnosed with mental defect or imbecility within the Lunacy Office archives, and none after 1949. This end point may well be a quirk of the process of selecting files for retention,^63^ but the small numbers overall suggest that alternative arrangements for the management of the property of those perceived as mentally deficient were often made, through informal mechanisms or during their childhood, without recourse to the Lunacy Office. Nevertheless, their inclusion in the workload of the Office contributed to changes in how mental capacity was understood – as something related to knowledge, understanding and independence of thought, rather than the absence of delusions. In Lunacy Office cases of mental defect, which had inherited from much earlier legal responses to ‘idiocy’ an implied incurability, incapacity was also understood as permanent. This shift was accelerated by accounts of ‘infirmity due to age’.

**Figure 1:**
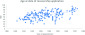
Patients’ ages (where known) when a receiver was first appointed for them, showing an upward trend.

The appointment of receivers because of ‘infirmity due to age’ became increasingly common from the 1920s, coinciding with anxiety about an ageing population. Unlike mental deficiency, there was no legislation directly affecting the citizenship status of older people, but they were presented as a drain or, in Pat Thane’s words, a ‘menace’, and some research from the time posited ‘a “blunting” of the senses and emotions’ in older people.^64^ Innovations in psychiatry were very slow to reach older patients, who were viewed as less likely to benefit.^65^ ‘Senile dementia’ took shape as a distinct diagnosis, and, as historian and psychiatrist Claire Hilton has argued, it was perceived as incurable, rapidly degenerative and an inevitable part of the normal ageing process until the 1960s and beyond.^66^ In this context, full citizenship for older people became more precarious. The ages of those whose files remain in the archives suggest a gradual upward shift in the age profile of Court of Protection patients, as shown in Figure 1. By 1962, the Master of the Court of Protection could say that the most common form of infirmity coming to the Court’s attention was ‘the mental confusion which so often accompanies old age, the difficulty of distinguishing the past from the present, and forgetfulness which leads to bills being left unpaid’.^67^

Typically, those who were incapable due to age were women over seventy, usually living in hospitals or residential nursing homes, although there were a small number of men and a few individuals who had the means to remain at home with round-the-clock care. Sarah Chabot, the first to be diagnosed with senile dementia in the Court of Protection archives, was representative: she was 78 years old when her daughter and son-in-law applied for a receiver for her in 1923, and she had recently moved into a nursing home. Her doctor described ‘marked loss of memory, perception disordered so that mistakes of identity occur, failure to locate where she is and how long she has been in a place, and fits of depression’. Her memory and perceptions of the world around her continued to deteriorate for some years, until she was more or less ‘helpless’.^68^

As with those categorised as ‘mentally deficient’, such descriptions highlighted knowledge deficits. Among older people, these were connected to memory loss. Mrs Chabot could not remember where she was or the identities of those around her. In 1931, wealthy and elderly Emily Willard could not recall at all what property she owned, and a few years later, 78-year-old Verner White had ‘no idea as to his Income or his liabilities and is unable to discuss his financial affairs intelligently’.^69^ Neither George Mowers nor Isabel Sykes, both in their 70s and diagnosed with dementia, could give any description of their property.^70^ These states, like mental defect, were not seen as amenable to treatment. Similar references to a lack of knowledge about money in general, and the individual’s property in particular, began to appear in the files of those who were neither mentally defective nor elderly and infirm. In 1965, for example, 45-year-old Lois Lewty was diagnosed with ‘chronic schizophrenia’ and found incapable of managing her affairs because she did not know ‘the amount of her annual income and says she is afraid to spend money because she is afraid that this will increase the National Debt. She does not understand Bank Statements, nor know where the Bank obtains the money to pay her cheques.’^71^ Although delusions and hallucinations still featured at times within the reports received by the Court of Protection (and both confusion and forgetfulness had appeared in earlier accounts too), ideas of what constituted or indicated incapacity narrowed. Descriptions of memory failure, disorientation for time and place, and gaps in knowledge came to dominate from the 1940s onwards. So too did references to senile dementia and its corollary for the under-70s, ‘pre-senile dementia’.

In light of this, it is perhaps unsurprising that from the 1950s the archives contain no examples of patients deemed cured and receiverships dissolved or of patients given partial control over their affairs. Mania, melancholia and delusions were disturbing and could lead to long-term institutionalisation, to be sure, but at least it was conceivable that the individual might benefit from some independence or even recover. For those with a diagnosis of mental defect or (pre-) senile dementia, being incapable of managing one’s affairs was more likely to be permanent, and the boundary between capacity and incapacity was more clear-cut. Ideas of citizenship also contributed to this narrowing of the Court of Protection’s focus, as will be discussed more fully in the next section. The growing welfare state and its emphasis upon full employment excluded those who did not or could not work, including those with more severe learning disabilities and the elderly. Within this framework, the elderly who were no longer able to work and who often still lived in institutions in the event of frailty or mental infirmity could be conceptualised more easily as less than full citizens, enabling ongoing interventions into their affairs through the Court of Protection and its decisions regarding their mental capacity. However, attention to the process and quality of decision-making among those with dementia or ‘mental defect’ also brought with it another way of seeing incapacity. Here, sources of concern were not the presence of delusions or a weakened intellect or memory, but an individual’s vulnerability.

## 3 1920s–40s: Attention to Vulnerability

Cases in which vulnerability took centre stage emerge in the 1920s and seem to disappear in the 1940s. Their arrival is relatively easy to explain in the context of the larger mental health and social welfare landscape, as well as the shift in Lunacy Office work outlined above. The interwar period was the high point of the mental hygiene movement, with its attention to borderline forms of mental weakness that might manifest as, or be caused by, maladjustment, emotional imbalance, delinquency, dysfunctional families and so on. The National Council for Mental Hygiene (founded in 1922) and Child Guidance Council (founded in 1927) encouraged early interventions from psychiatrists, psychologists, psychiatric social workers and even GPs and teachers in the name of national (economic) health. The community as a whole, and not just the individual, became a potential site for mental disease, and social relationships were often the source of both illness and cure.^72^ Attention on the part of the Lunacy Office to a prospective patient’s social circumstances and interactions, as well as the more general fact of its willingness to intervene in these borderline cases, makes sense as part of this.

For those whose capacity was considered in terms of their vulnerability, questions of illness were unimportant. They had either fully recovered from an illness or had not been diagnosed with any disorder or illness at all. Even so, their incapacity was not fully decoupled from clinical conditions: medical opinion was always sought, and doctors often borrowed the language of mental defect and senile dementia in their accounts. This language tended to conceal the central issue, which was whether an individual could act autonomously in the management of their affairs, ‘not being a slave to instinct, or whim or caprice’, but making decisions that were authentically their own.^73^ Such decisions could be distorted not only by instinct, whim or caprice but also by the malign influence of other people, including family members, neighbours and those presenting themselves as friends or even suitors. The vital question was whether the individual was susceptible to such influence, although vulnerability itself was not mentioned. Social circumstances, personalities and support networks were all explicitly considered, while age, gender and class informed the conclusions that the Lunacy Office and its informants reached. In these cases, individuals were not seen in any comprehensive sense as wholly incapable: a firm distinction between capacity and incapacity was absent. Indeed, individuals were often granted considerable independence and control over their affairs. Nevertheless, the questioning of their capabilities and the decisions to place their property under the control of receivers runs hand-in-hand with perceptions of these individuals as slightly less than full citizens.

Three case studies will help to illustrate these points. These are not typical examples of the work of the Lunacy Office or its archive: these receiverships were either contested or eventually dissolved. This was rare but useful, since it called for a more explicit consideration of why an individual was to be considered incapable. The first is that of Arthur Short, whose capabilities were called into question in anticipation of his twenty-first birthday.^74^ Mr Short had enjoyed an eventful adolescence in the 1910s, firstly suffering a ‘disease of the glands’ and a period of ‘acute insanity’ before joining the army at sixteen with a lie about his age, spending money rather extravagantly and being court martialled. His mother then arranged an apprenticeship at a garage for him and supported him financially. He stood to inherit from his late father when he came of age in 1921, when he would receive an income of around £270 a year (around £12,500 today). Mr Short supported the application for his mother to act as his receiver, saying as much to the doctors who examined him and even writing to the Lunacy Office to state that it would ‘relieve me of the responsibility’ of worrying about money.

The original application included both a medical affidavit and statement from Mr Short’s employer, but the medical affidavit was deemed ‘too thin’ by the Lunacy Office. This was perhaps unsurprising: it dwelt on his lack of interest in cars, twitching fingers, irritability and persistent yawning. However unconvincing this was as an account of incapacity, Lunacy Office officials were still willing to find a way to intervene, and they requested the views of one of the Lord Chancellor’s visitors. This expert, Sir James Crichton-Browne, was able to muster a specific diagnosis. Sir James’s advanced years enabled him to draw upon a very nineteenth-century diagnosis. Finding Mr Short to be ‘intelligent in conversation, quite rational, of good memory of pleasing presence and free from delusions’, he diagnosed ‘moral insanity’ and, later in the report, ‘moral and mental defect’. Key to this was Mr Short’s ‘inability to distinguish between right and wrong and lack of self control’. As well as misconduct while in the army, Crichton-Browne cited Mr Short’s thefts from his employer and willingness to admit both this and his habits of going to pubs and ‘walking out with a girl whom he picked up in the street’. Crichton-Browne gave greater weight to Mr Short’s lack of shame than his mild difficulties with arithmetic, but to bolster his opinion, the eminent expert echoed discussions of mental deficiency with a suspicion that the childhood illness had probably ‘damaged his brain’. On the strength of this, Mr Short’s mother was appointed as his receiver.

Mr Short and his affairs proved somewhat troublesome for the Office. Mr Short himself was often in touch, asking for money for anything from business investments to next week’s rent. He asked if he could move to Australia and if he could get married. The Lunacy Office viewed Mr Short’s plans for business investment and working abroad, which had been suggested to him by third parties, with a distinct lack of enthusiasm. There was also concern about tensions in the Short family and the influence (or lack thereof) exerted over Mr Short by his uncles. In the eyes of the Office, he was isolated from positive influences, particularly male influences, and surrounded by acquaintances full of unwise ideas.

As the years passed, Mr Short became increasingly annoyed by the involvement of the Office in his affairs and often raised the possibility of its termination. The visitors who saw him every year were divided in their views: Lord Sandhurst reported that he ‘observed nothing to differentiate him from many other young men whose affairs have not been taken out of their hands’, and that it would be ‘difficult to support an application for appointment of a Receiver if the matter were Res nova’. Dr Raw found him ‘irresponsible’, ‘impulsive and uncertain in his behaviour’ and, later, ‘quite rational but perhaps a little childish’. Barrister Hubert Meysey Thompson described him in florid terms as ‘loquacious, grandiloquent and aggrieved’, but thought that he was probably capable of managing his affairs and might even benefit from being given the chance to do so. Even during a four-year period of hospitalisation following an attempted suicide, reports continued the same theme of uncertainty. Mr Short appeared to be rational and intelligent (and, indeed, ‘a fine looking man’) but also extremely irritable and troublesome, continually showing little self-control and getting into trouble at every turn. Yet, soon after being discharged from hospital in late 1938, Mr Short finally submitted his long-promised application to terminate the receivership, and in April 1939 he was restored to his property.

From the outset of his case, Arthur Short was acknowledged as bordering on capable of managing his affairs. He occupied an uncertain space between capacity and incapacity, where the Office felt able to intervene through a receivership but permitted Mr Short conduct of his own employment, earnings, tenancies and other day-to-day decisions about money and affairs. A definitive diagnosis, complete with references to brain damage, plus his own support for the proposal were both necessary for the appointment of a receiver. During the receivership, though, references to mental defect, moral insanity or brain damage disappeared: those who felt that Mr Short should not have control over his property based their views upon his reported irresponsibility and his tendency to react quickly to those around him – either with irritability or by falling in too readily with their ideas and suggestions. He was vulnerable to the negative influence of others and in the face of his own lack of forethought and self-control. As a young, lower-class man, with a history of delinquency, his potential independence was a worry, but one that could be resolved (for a while at least) by limiting his financial independence and appointing a receiver. From the 1950s, as Abigail Wills has shown, youth delinquency was viewed as a breach of the duties of citizenship;^75^ perhaps in the case of Arthur Short we see an earlier example of youth and minor misconduct casting an individual as a less than complete citizen.

For Miss Jean Carr, it was ostensibly her memory, not her conduct, which rendered her incapable.^76^ This aligned her with the emerging group of Lunacy Office patients diagnosed with ‘senile dementia’. Miss Carr, though, was barely 21. She had suffered from a ‘functional disorder of the nervous system’ as a teenager in the 1920s, and although this was no longer a factor in her well-being, she was said by her mother to be ‘delicate’ with little experience of ‘social affairs’ as she approached the age of majority. Her medical attendant provided a full report of her difficulties, which focused on memory:

inability to concentrate on any subject for more than a few minutes at a time. E.g. she will start having a meal and forget to go on with it requiring constant urging. When spoken to on any subject she will wander on to some other in a minute or two. If about to brush her teeth will forget what she is going to do. If writing a letter will start it but is unable to continue. Will put her shoes in a cupboard and forget where they are.

The Lunacy Office, swayed in all likelihood by the extremely large fortune that Miss Carr would soon inherit, found that she was incapable of managing her affairs and appointed a receiver for her in 1934. As time passed, though, it became clear that other factors were being weighed in the balance too.

Miss Carr became increasingly annoyed at her lack of independence and applied on several occasions to have the receivership lifted. This involved gathering support from her doctors and discussing the matter in detail with the visitors who came to see her on a regular basis, including sometimes at her special request. The question was considered and rejected three times before the receivership was finally terminated in December 1940. Learning from experience, Miss Carr and her doctors had provided the Office with overwhelming evidence on this last occasion, affirming not simply that she was capable, but that she was confident, intelligent and business-like in her approach to her affairs. Reluctantly, the Assistant Master accepted that he had no grounds to refuse.

This reluctance to dissolve the receivership is striking. At its root lay concerns that Miss Carr was vulnerable due to her isolation and lack of self-confidence and experience, leaving her exposed to the machinations of others and particularly any unscrupulous suitor or husband that might appear on the scene. Her lack of worldly knowledge and the spectre of potential husbands with ill intent were raised in reports, in response to her requests to be restored to her property and in file notes, and were undoubtedly informed by the fact that she was young, female and in possession of a very large fortune as a result of her father’s death. There were also reports of family tensions, particularly between mother and daughter and then between Miss Carr and her brother, hinting that any influence wielded by those around Miss Carr with an interest in preserving the family fortune might be fleeting at best.

With these considerations in mind, the Office responded to her first application for restoration with a compromise: the receivership would remain, but her income would be paid into a bank account in Miss Carr’s name, for her own use absolutely. This came to £65 a month (something like £4000 per month today): a large sum. The Office also enquired whether she would be willing to enter into a voluntary settlement with a view to protecting her capital, particularly from an unprincipled husband. The Office perceived Miss Carr as possessing legal capacity and the ability to make certain decisions about her affairs but *also* in need of protection, rather than simply and absolutely incapable.

**Figure 2:**
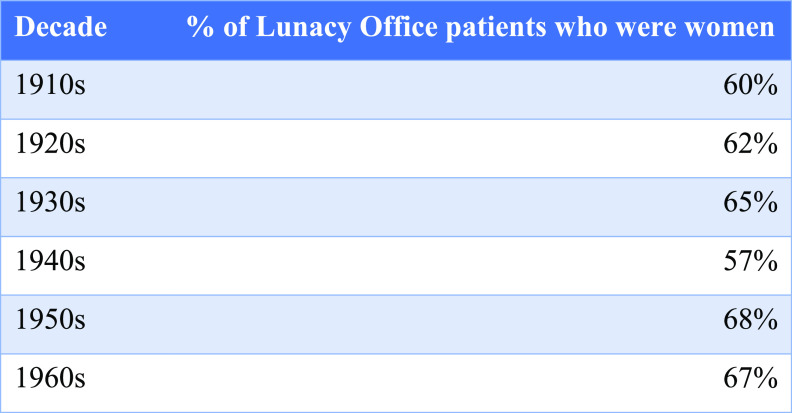
Women consistently made up more than half of all Lunacy Office patients.

As someone young, affluent, female and without father or father figure, Miss Carr’s independence was the source of significant concern, and those around her displayed a clear readiness to curtail her citizenship through the Lunacy Office. It is notable that vulnerability was more readily perceived in women than in men. There was consistently a larger proportion of women than men among Lunacy Office clients, as shown in Figure 2, and Mr Short’s file is the only example in the archives of attention to vulnerability in the case of a man. Anxiety about women’s roles and women’s independence in the interwar period may have played a part here. Historians have debated the impact of the First World War upon women and ideas of gender, but the extended franchise, higher rates of employment for women outside of the home and rising numbers of unmarried or widowed women undoubtedly prompted anxiety in some quarters.^77^ Were women sufficiently mature and stable for these new roles and responsibilities? Did their pursuit of independence present problems? At the same time, state interventions in the form of welfare provision began to assume women’s dependence upon men, effectively excluding women from ‘full social citizenship’. Wartime allowances and widows’ pensions, for example, implied that women ‘merited concern only as the residual legatees of their citizen-husbands’, and saw the state step in as surrogate husband (or father) where such male influence was absent.^78^ Women were not quite full citizens, and the extent and quality of their citizenship was embroiled with perceptions of their autonomy. As Miss Carr’s case suggests, there was perhaps a greater readiness on the part of those who initiated contact with the Lunacy Office – relatives, solicitors, neighbours and friends – as well as Office officials themselves to be concerned about women’s decision-making and to perceive them as vulnerable, unable to negotiate the demands and pressures of independent living and in need of state oversight where no husband or father was available to take responsibility for their affairs.

My third and final case study provides another example of concern over a woman’s decision-making: in this case, an older and much less wealthy woman. We return to Chilfrome, The Old Rectory and Miss Beatrice Alexander.^79^ Less than two weeks after the Lunacy Office was alerted to her case by Dr Stephenson, a neighbour and friend of Miss Alexander’s late benefactor, Miss Alexander received a visit from one of the Lord Chancellor’s visitors. What he found displeased him as much as it did Dr Stephenson: Miss Alexander’s weekly income seemed to go straight to the Humphries family, many of whom lived in The Old Rectory. The garden was not well kept, he noted, even though Mr Humphries had previously worked there as a gardener. Mrs Humphries was ‘a coarse looking woman with the appearance of a heavy drinker. She was by turns truculent and cringing and tried to stop me talking to Miss Alexander alone,’ he recorded. Miss Alexander he found ‘a pleasant little lady’ but one who was vague about her affairs. Though there was no definite evidence that the Humphries were ‘robbing or neglecting’ Miss Alexander, the situation was ‘most irregular and unsatisfactory’, and the visitor had no doubt that a receiver should be appointed as soon as possible.

The application for the appointment of the Official Solicitor as receiver did not proceed smoothly. A solicitor appeared on behalf of Miss Alexander to contest the appointment, using the statement of Dr Margaret Vivian of Bournemouth, who had found Miss Alexander ‘of sound mind and quite capable of managing her own affairs’. Dr Vivian acknowledged that Miss Alexander’s ‘reactions were somewhat slow’, but maintained that her answers were intelligent and that she appeared to appreciate the value of money and the importance of paying accounts on time. Intellect and specific knowledge were deployed as evidence of mental capacity, but Lunacy Office officials and Dr Stephenson took the very existence of this medical report as further evidence of the Humphries’ influence over Miss Alexander’s life, assuming that the Humphries had engineered it. Acknowledging that the existing medical evidence of incapacity was light, the Master requested that one of the Lord Chancellor’s *medical* visitors visit Miss Alexander and provide a report. Although a copy of this report does not survive, it was sufficient for the Office to proceed, and the Official Solicitor took control of Miss Alexander’s affairs in late summer 1939.

By April 1940, after a tumultuous period at The Old Rectory and her removal to the town of Broadstairs while the Humphries were evicted, Miss Alexander returned home with her new nurse-companion, Kate Wortt. Miss Wortt described her charge as ‘very highly strung & nervous’ but not in any way mentally weak or abnormal. Indeed, she was ‘well read & clever & can talk well on many subjects, three separate Drs have seen her & in every case the verdict is highly strung but not mental’. In Miss Wortt’s eyes, ‘the only thing I find with her is, she can be dominated by a stronger will’. Like the official visitors and the neighbour Dr Stephenson, Miss Wortt saw Miss Alexander as vulnerable around other more confident and less well-meaning people, who could influence her to make decisions that might not reflect her own wishes. Left to fend for herself, Miss Alexander could not resist such influence and maintain her autonomy, and so she was not capable of managing her own affairs. With Miss Wortt’s positive influence, and the receivership as a final safeguard, all agreed that a satisfactory situation had been achieved – even, we might tentatively think, Miss Alexander herself.^80^

Given her comfortable home and income, her lack of experience with such property, and the absence of any trusted family or friends around her, Miss Alexander was seen as particularly susceptible to the interference of those who might take advantage. Like Miss Carr, she was without husband or father, but while Miss Carr could gain experience and confidence as she matured, Miss Alexander’s perceived deficits may have been connected to her social status as a retired housekeeper and her personality, rather than her age. Despite his reference to her ‘low mentality’, Dr Stephenson had put his view most clearly when he described her as ‘without character and without courage’. This was echoed in visitors’ accounts of her as pleasant but easily overwhelmed. Her character, her inexperience and the circumstances in which she found herself in her later years all rendered her vulnerable to the unkind influence of others. Her incapacity was something of a grey area, but by intervening the Office showed a willingness to consider this kind of vulnerability, not only delusion or intellectual impairment, and to locate it in women.

It is worth adding that the centrality to these cases of vulnerability was not overtly acknowledged. This echoes the argument made by Margaret Hall in relation to contemporary Canadian guardianship decisions, where she finds that vulnerability is at the heart of how cases are decided but is not recognised as such, leading to unproductive debate about individual autonomy versus protection.^81^ This same failure in the mid-twentieth century Lunacy Office to recognise the importance of both vulnerability and the idea of citizenship to their decision-making may have enabled the emergence of a firmer delineation between capacity and incapacity in the 1940s and 1950s, when cases such as those of Miss Carr, Mr Short and Miss Alexander disappeared. Without being clear about the issues at stake when decisions about incapacity were considered, it was easier to overlook changes or inconsistencies over time in how such decisions were being made.

This lack of attention to change was exacerbated by the absence of external pressure on the Court to change its practices; there was no apparent desire to restrict its reach. Despite growing concern in medical and social work, the voluntary sector and the media about the treatment and the rights of those institutionalised as a result of mental deficiency, old age or mental illness, the activities of the Court of Protection attracted no negative attention.^82^ Evidence to the Percy Committee in the 1950s said little on the subject, but what it did say was gently supportive of extending rather than limiting the Court’s scope. The Royal Medico-Psychological Association, for example, recommended *easier* access to financial protection for patients (voluntary or otherwise) as soon as they were admitted to hospital,^83^ and this was the position adopted in mild terms by the Committee in its final report.^84^ The work of the Court was barely touched by the final terms of the 1959 Mental Health Act. Decisions to place an individual’s property in the hands of a receiver remained entirely uncontroversial and unnoticed until the drafting of the 2005 Mental Capacity Act.

In the absence of external pressures for change, or internal debate about the proper boundaries of mental capacity, how might we explain the disappearance of vulnerability and the rise of specific knowledge deficits within Court of Protection files? Here, attention to citizenship is useful once again. As Mathew Thomson and others have argued, emphasis in the years following the Second World War upon social responsibility, employment and universality as part of the growing welfare state had a variable effect upon the status of those with disabilities.^85^ Perhaps counter-intuitively, the expansion of state welfare did not encourage greater intervention on the part of the Court of Protection but the opposite. The principles of universality did not sit comfortably with the idea of an individual like Miss Carr or Miss Alexander being sometimes vulnerable and somewhat incapable. Mental incapacity had to be conceptualised more clearly as something qualitatively different from the state of the ‘ordinary’, competent citizen. Bespoke solutions, in which partial or circumstantial incapacity was permitted, were also more difficult to design within the contours of a large welfare state, in which individual status (as mother, worker, OAP) tended towards the absolute. And on a practical note, the provision of free healthcare removed the incentive for local authorities, previously interested in a hospital patient’s property for the payment of fees, to make contact with the Court of Protection on behalf of the mentally infirm being treated within their boundaries.^86^ In the 1940s and 1950s, then, a hard boundary between those who were capable and those who were not began to form. Cases where illness was absent but vulnerability was identified disappeared, and the increase in receiverships dating from the 1890 Lunacy Act quietly ceased.

## 4 Conclusions

The Court of Protection may have attracted little official or external scrutiny for most of the twentieth century, but that does not mean that its activities were insignificant, unchanging or unremarkable. It was empowered to take drastic action, declaring an individual incapable of managing their own affairs and placing their property and decision-making in the hands of another. Its approach to this in practice was, at times, more nuanced than some popular impressions of its history might suggest. Mental incapacity was not always conceptualised in stark or simplistic terms, and what constituted evidence of incapacity (or cause for enquiry into incapacity) has changed. The Court’s archives demonstrate that there were shifting templates for incapacity, from the delusional individual detained under certificate to the confused elderly woman diagnosed with senile dementia. For a time, its personnel also showed themselves willing, by paying attention to vulnerability, to consider a wider variety of individuals as needing protection and help. This directs our attention to some of the ways in which incapacity has been perceived in the past in relation to gender, age and citizenship.

This picture is incomplete: the archives have relatively little to say about perceptions of incapacity among lay people, geared as they are towards valuing the assessments of medico-legal professionals. They are also largely silent from the 1960s onwards, leaving a gap still to be bridged from the post-war years to the sudden arrival of mental capacity as a vital issue for medical law in the 1990s. Further research, considering case law and advocacy work, will no doubt add to this story, and there is likely more to be said about attitudes towards citizenship (or rights) and mental capacity decisions. Looking beyond these questions, the archives themselves are rich in other ways that are yet to be explored. They are surprisingly revealing of the everyday experiences of those found to lack capacity. Less surprisingly, given that the records are propelled by emotive issues of health, money and family, they are also full of human drama and tragedy, bad luck and betrayal, loyalty and love. They offer a salutary reminder of the relationship between medico-legal ideas and the social spheres in which they take shape, and of the individual stories like Miss Alexander’s that lie behind our discussions of concepts and trends.

## Footnotes

1.
In the National Archives in London (hereafter TNA), three files relating to Miss Alexander are currently open: J127/24 [1939], J127/25 [1939/40] and J127/26 [1940].

2.
Section 90 (1) of the Lunacy Act 1890. The Act is reproduced in full in S.G. Lushington, *Archbold’s Lunacy*, 4th edn (London: Shaw & Sons, 1895).

3.

T.C.S. Keely , ‘One Hundred Years of Lunacy Administration’, The Cambridge Law Journal, 8, 2 (1943), 198; Gerald E. Mills and Ronald W. Poyser, *Lunacy Practice* (London: Butterworth & Co, 1934), v; The Law Commission, *Mental Incapacity: Mentally Incapacitated Adults* (London: HMSO, 1995), 10.

4.
As this article covers the work of this Office before and after the change of name, I will refer to both the [Lunacy] Office and the Court of Protection. For the avoidance of doubt, they are one and the same thing.

5.
Toby Williamson interviewed by Dr Mark Porter on ‘Weaning Babies, Seeing the Same Doctor Saves Lives, NHS Research, Mental Capacity’, *Inside Health*, produced by Fiona Hill, BBC Radio 4, first broadcast 17 July 2018.

6.
This perspective among new judges was highlighted by former Senior Judge Denzil Lush in a lecture delivered in November 2018 to the Association of Contentious Trust and Probate Specialists.

7.
Work on earlier periods includes Ezra Hasson, ‘Capacity to Marry: Law, Medicine and Conceptions of Insanity’, *Social History of Medicine*, 23 (2009), 1–20; Mark Jackson, “‘It Begins with the Goose and Ends with the Goose”: Medical, Legal, and Lay Understandings of Imbecility in Ingram v Wyatt, 1824–32’, *Social History of Medicine*, 11 (1998), 361–80; Simon Jarrett, ‘‘‘Belief”, “opinion”, and “knowledge”: the idiot in law in the long eighteenth century’, in Patrick McDonagh, C.F. Goodey, and Timothy Stainton (eds), *Intellectual Disability: A Conceptual History, 1200–1900* (Manchester: Manchester University Press, 2018), 162–89; Chantal Stebbings, ‘Protecting the Property of the Mentally Ill: The Judicial Solution in Nineteenth Century Lunacy Law’, *The Cambridge Law Journal*, 71, 2 (2012), 384–411; Akihito Suzuki, *Madness at Home: The Psychiatrist, the Patient and the Family in England, 1820–60* (Berkeley, CA: University of California Press, 2006).

8.
Peter Bartlett, ‘Sense and nonsense: sensation, delusion and the limitation of sanity in nineteenth-century law’, in Lionel Bently and Leo Flynn (eds), *Law and the Senses: Sensational Jurisprudence* (London: Pluto Press, 1996), 21–41; Peter Bartlett and Ralph Sandland, *Mental Health Law: Policy and Practice*, 1st edn (London: Blackstone Press, 2000), 349.

9.
For example, Angharad E. Beckett, *Citizenship and Vulnerability: Disability and Issues of Social and Political Engagement* (Basingstoke: Palgrave Macmillan, 2006); Susan Pedersen, ‘Gender, Welfare, and Citizenship in Britain during the Great War’, *The American Historical Review*, 95, 4 (1990), 983–1006; Mathew Thomson, *The Problem of Mental Deficiency: Eugenics, Democracy and Social Policy in Britain, c.1870–1959* (Oxford: Clarendon, 1998); Jonathan Toms, ‘Citizenship and Learning Disabled People: The Mental Health Charity MIND’s 1970s Campaign in Historical Context’, *Medical History*, 61, 4 (2017), 481–99.10.1017/mdh.2017.55PMC562960628901871

10.
Kathleen Jones, ‘Law and mental health: sticks or carrots?’, in German E. Berrios and Hugh Freeman (eds), *150 Years of British Psychiatry, 1841–1991* (London: Gaskell, 1991), 89–102; Kingsley Jones, ‘The Windham Case: The Enquiry Held in London in 1861 into the State of Mind of William Frederick Windham, Heir to the Felbrigg Estate’, *British Journal of Psychiatry*, 119, 551 (1971), 425–33; Clive Unsworth, *The Politics of Mental Health Legislation* (Oxford: Clarendon, 1987).10.1192/bjp.119.551.4254942958

11.
As characterised in Phil Fennell, *Treatment without Consent: Law, Psychiatry and the Treatment of Mentally Disordered People since 1845* (London: Routledge, 1991), 12.

12.
For example, David Armstrong, *Political Anatomy of the Body: Medical Knowledge in Britain in the Twentieth Century* (Cambridge: Cambridge University Press, 1983); Nikolas S. Rose, *Inventing Our Selves: Psychology, Power and Personhood* (Cambridge: Cambridge University Press, 1998).

13.
Beverley Clough, ‘Disability and Vulnerability: Challenging the Capacity/Incapacity Binary’, *Social Policy and Society*, 16, 3 (2017), 469–81; Margaret I. Hall, ‘Mental Capacity in the (Civil) Law: Capacity, Autonomy and Vulnerability’, *McGill Law Journal*, 58, 1 (2012), 61–94.

14.
Section 116 (d) of the Lunacy Act 1890.

15.
TNA J92/10 [1911–39].

16.
TNA J92/20 [1915–42].

17.
Bartlett and Sandland, *op. cit.* (note 8), 341.

18.
TNA J92/211 [1949–83]. Additional examples are TNA J92/89 [1938/39] and TNA J92/233 [1951–81].

19.
TNA J92/29 [1918–39].

20.
Mills and Poyser, *op. cit.* (note 3), p. 16. This was not the case in the earlier edition of the book, from 1927.

21.
Joseph Elmer, *The Practice in Lunacy under Commissions and Inquisitions*, 6th edn (London: Stevens & Sons, 1877), 9. For more on inquisitions, which had largely disappeared by the early twentieth century, see Peter Bartlett, ‘Legal Madness in the Nineteenth Century’, *Social History of Medicine*, 14, 1 (2001), 107–31; Jarrett, *op. cit.* (note 7); Stebbings, *op. cit.* (note 7); Suzuki, *op. cit.* (note 7).10.1093/shm/14.1.10714524353

22.
Elmer, *op. cit.* (note 21), 128, 129.

23.
Bartlett and Sandland, *op. cit.* (note 8), 341.

24.

*Report of the Royal Commission on the Care and Control of the Feeble-Minded* (London: HMSO, 1908), 259.

25.

*Royal Commission on the Law Relating to Mental Illness and Mental Deficiency 1954–57: Report* (London: HMSO, 1957), 291.

26.
On changing views of mental illness and treatment, see Akinobu Takabayashi, ‘Surviving the Lunacy Act of 1890: English Psychiatrists and Professional Development during the Early Twentieth Century’, *Medical History*, 61, 2 (2017), 246–69; Louise Westwood, ‘Explorations of Scottish, German, and American psychiatry: the work of Helen Boyle and Isabel Hutton in the treatment of noncertifiable mental disorders in England, 1899–1939’, in Volker Roelck, Paul Weindling and Louise Westwood (eds), *International Relations in Psychiatry: Britain Germany, and the United States to World War II* (Rochester, NY: University of Rochester Press, 2010), 179–96.10.1017/mdh.2017.4PMC542630428260566

27.
On criminal law: Arlie Loughnan, *Manifest Madness: Mental Incapacity in Criminal Law* (Oxford: Oxford University Press, 2012); Janet Weston, *Medicine, the Penal System and Sexual Crimes in England, 1919–60s: Diagnosing Deviance* (London: Bloomsbury Academic, 2017).

28.

*Report on the Law Relating to Mental Illness*, *op. cit.* (note 25), 292; Donald G. Hunt and Maurice E. Reed, *Heywood & Massey’s Court of Protection Practice*, 8th edn (London: Stevens & Sons, 1961), vi.

29.
Raymond Jennings, ‘Mental Disorder and the Court of Protection’, *The Lancet*, 279 (1962), 855–6.10.1016/s0140-6736(62)91865-214451616

30.

*Report on the Law Relating to Mental Illness*, *op. cit.* (note 25), 291.

31.
TNA J92/114 [1935–82].

32.
For some typical examples, see TNA J92/114 [1935–82] and TNA J92/84 [1936–40].

33.

*Report on the Law Relating to Mental Illness*, *op. cit.* (note 25), 292.

34.
TNA J92/199 [1947–83].

35.
For more on the Lord Chancellor’s visitors, see Michael Neve and Trevor Turner, ‘What the Doctor Thought and Did: Sir James Crichton-Browne (1840–1938)’, *Medical History*, 39 (1995), 399.10.1017/s002572730006035xPMC10370288558991

36.
TNA J92/29 [1918–39].

37.
TNA J92/100 [1927–82].

38.
TNA J92/116 [1935–82].

39.
TNA J92/59 [1928–41]; J92/63 [1929–39]. See also TNA J92/80 [1935–40].

40.
TNA J92/94 [1939].

41.
TNA J92/38 [1921–39]; J92/77 [1934–41].

42.
For example, TNA J92/54 [1926–41], J92/83 [1936–39] and J92/97 [1924–81]. See also J92/3 [1902–39], where the receivership continued.

43.
TNA J92/19 [1914–40]; J92/67 [1930–41].

44.
TNA J127/227 [1964–76].

45.
TNA J92/54 [1926–41]; TNA J92/12 [1912–40]; TNA J92/38 [1921–39]. See also TNA J92/19 [1914–40], TNA J92/77 [1934–41] and TNA J92/67 [1930–41].

46.
TNA J127/39 [1921–41]; TNA J92/29 [1918–39]; TNA J92/41 [1922–40]. For an exception to the rule, Marriner Kemp was not consulted at all: TNA J92/42 [1922–42].

47.
See the comments in, for example, Henry Studdy Theobald, *The Law Relating to Lunacy* (London: Stevens & Sons, 1924), 42, and later textbooks.

48.
Mills and Poyser, *op. cit.* (note 3), 16.

49.
Theobald, *op. cit.* (note 47), 5–6.

50.
Beverley A. Clough, ‘New Legal Landscapes: (Re)Constructing the Boundaries of Mental Capacity Law’, *Medical Law Review*, 26, 2 (2018), 246–75: 255.10.1093/medlaw/fwy01229618100

51.

*Report on the Care and Control of the Feeble-Minded*, *op. cit.* (note 24), 7.

52.
Mental capacity in the context of personal welfare decisions was defined in *Re C (Refusal of Medical Treatment)*[1994] 1 WLR 290. The meaning of being ‘incapable to manage one’s affairs’ was finally tested in *Masterman-Lister v Brutton & Co* [2002] EWHC 417 (QB) and [2002] EWCA Civ 1889. Thanks to Denzil Lush for bringing this latter case to my attention.

53.
James Munby, ‘Capacity: A Lawyer’s Perspective’, *Medico-Legal Journal*, 80 (2012), 69. See also *Report on the Law Relating to Mental Illness*, *op. cit.* (note 25), 290. The exception to this is testamentary capacity, for which a four-part test was established in *Banks v Goodfellow* in 1870.10.1258/mlj.2012.01201322761360

54.
Bartlett and Sandland, *op. cit.* (note 8), 249.

55.
For example, TNA J92/42 [1922–39]. Reportedly imagined illnesses are also recorded in TNA J92/22 [1915–42], J92/76 [1933–40], and crimes in TNA J92/3 [1902–39], J92/115 [1935–80].

56.
Bartlett and Sandland, *op. cit.* (note 8), 249.

57.
Pamela Dale and Joseph Melling, *Mental Illness and Learning Disability since 1850: Finding a Place for Mental Disorder in the United Kingdom* (London: Routledge, 2006); Mark Jackson, *The Borderland of Imbecility: Medicine, Society, and the Fabrication of the Feeble Mind in late Victorian and Edwardian England* (Manchester: Manchester University Press, 2000); Harvey G. Simmons, ‘Explaining Social Policy: The English Mental Deficiency Act of 1913’, *Journal of Social History*, 11, 3 (1978), 387–403; Thomson, *op. cit.* (note 9).10.1353/jsh/11.3.38711632372

58.
Thomson, *op. cit.* (note 9), 34.

59.
TNA J92/14 [1913–41].

60.
TNA J92/12 [1912–40]; TNA J92/207 [1948–81]; TNA J92/159 [1944–80].

61.
TNA J92/12 [1912–40].

62.
TNA J92/57 [1927–41]; TNA J92/61 [1928–41].

63.
Many later files were closed in 1983, when unclaimed balances held by the Court of Protection – often relating to patients who had died decades earlier – were investigated. Unclaimed balances may have occurred more frequently among elderly patients outliving or losing touch with next of kin than among those classed as ‘mentally defective’.

64.
Charlotte Greenhalgh, *Aging in Twentieth-Century Britain* (Berkeley, CA: University of California Press, 2018), chapter 1; Pat Thane, *Old Age in English History: Past Experiences, Present Issues* (Oxford: Oxford University Press, 2000), chapter 17.

65.
Claire Hilton, *Improving Psychiatric Care for Older People: Barbara Robb’s Campaign 1965–75* (Cham: Palgrave Macmillan, 2017), 26.

66.
Claire Hilton, ‘Psychiatrists, Mental Health Provision and “Senile Dementia” in England, 1940s–79’, *History of Psychiatry*, 26, 2 (2015), 182–99; Duncan Wilson, ‘Quantifying the Quiet Epidemic: Diagnosing Dementia in Late 20th-Century Britain’, *History of the Human Sciences*, 27, 5 (2014), 126–46.

67.
Jennings, *op. cit.* (note 29).

68.
TNA J92/44 [1923–38].

69.
TNA J92/92 [1939–40].

70.
TNA J92/293 [1966–74]; J127/188 [1967–72].

71.
TNA J127/221 [1973–75].

72.
Armstrong, *op. cit.* (note 12); Courtauld Thomson, ‘A National Council for Mental Hygiene’, *British Medical Journal*, 1, 3196 (1922), 538; Jonathan Toms, *Mental Hygiene and Psychiatry in Modern Britain* (Basingstoke: Palgrave Macmillan, 2013).

73.
Derek Morgan, *Issues in Medical Law and Ethics* (London: Cavendish Publishing, 2001), 87.

74.
TNA J92/38 [1921–39].

75.
Abigail Wills, ‘Delinquency, Masculinity and Citizenship in England 1950–70’, *Past & Present*, 187, 1 (2005), 157–85.

76.
TNA J92/77 [1934–41].

77.
For a summary of the debate, see Adrian Bingham, ‘“An Era of Domesticity”? Histories of Women and Gender in Interwar Britain’, *Cultural and Social History*, 1, 2 (2004), 225–33.

78.
Pedersen, *op. cit.* (note 9), 1006.

79.
Miss Alexander’s case has some similarities with a much more recent case heard in the High Court: *London Borough of Redbridge v G*[2014] EWHC 485 (COP).

80.
It is difficult to be conclusive, particularly given the closure of later files and the paucity of correspondence from Miss Alexander, but reports from various parties describe her as happy and contented after returning to The Old Rectory, the house she very much loved and where she remained until her death. She is buried in the graveyard neighbouring The Old Rectory’s grounds.

81.
Hall, *op. cit.* (note 13).

82.
For example, Kenneth Hazell, *Social and Medical Problems of the Elderly* (London: Hutchinson Medical Publications, 1960); *50 000 Outside the Law: An Examination of the Treatment of those Certified as Mentally Defective* (London: National Council for Civil Liberties, 1951); *Care and Treatment of the Elderly* (London: British Medical Association, 1949).

83.

*Royal Commission on the Law Relating to Mental Illness and Mental Deficiency: Minutes of Evidence, 8th Day* (London: HMSO, 1954), 277.

84.

*Report on the Law Relating to Mental Illness*, *op. cit.* (note 25), 293.

85.
Thomson, *op. cit.* (note 9), 293.

86.
A point made by Sir Ronald Poyser in evidence to the Percy Commission: *Minutes of Evidence, op. cit.* (note 83), 1196.

